# Angiopoietin-2/-1 ratios and MMP-3 levels as an early warning sign for the presence of giant cell arteritis in patients with polymyalgia rheumatica

**DOI:** 10.1186/s13075-022-02754-5

**Published:** 2022-03-07

**Authors:** Yannick van Sleen, Philip Therkildsen, Berit Dalsgaard Nielsen, Kornelis S. M. van der Geest, Ib Hansen, Peter Heeringa, Marcel D. Posthumus, Maria Sandovici, Erik J. M. Toonen, Jannik Zijlstra, Annemieke M. H. Boots, Ellen-Margrethe Hauge, Elisabeth Brouwer

**Affiliations:** 1 Department of Rheumatology and Clinical Immunology, University Medical Center Groningen, Hanzeplein 1, Groningen, 9700 RB the Netherlands; 2 Department of Rheumatology, Aarhus University Hospital, Aarhus N, Denmark; 3 Department of Medicine, Horsens Regional Hospital, Horsens, Denmark; 4 Department of Pathology and Medical Biology, University Medical Center Groningen, Groningen, the Netherlands; 5 Department of Orthopaedic Surgery, Martini Hospital, Groningen, the Netherlands; 6 R&D Department, Hycult Biotechnology, Uden, the Netherlands

**Keywords:** Giant cell arteritis, Polymyalgia rheumatica, Look-alike patients, Angiopoietin-2, MMP-3, Platelets

## Abstract

**Background:**

Diagnosing patients with giant cell arteritis (GCA) remains difficult. Due to its non-specific symptoms, it is challenging to identify GCA in patients presenting with symptoms of polymyalgia rheumatica (PMR), which is a more common disease. Also, commonly used acute-phase markers CRP and ESR fail to discriminate GCA patients from PMR and (infectious) mimicry patients. Therefore, we investigated biomarkers reflecting vessel wall inflammation for their utility in the accurate diagnosis of GCA in two international cohorts.

**Methods:**

Treatment-naïve GCA patients participated in the Aarhus AGP cohort (*N* = 52) and the Groningen GPS cohort (*N* = 48). The AGP and GPS biomarker levels and symptoms were compared to patients presenting phenotypically as isolated PMR, infectious mimicry controls and healthy controls (HCs). Serum/plasma levels of 12 biomarkers were measured by ELISA or Luminex.

**Results:**

In both the AGP and the GPS cohort, we found that weight loss, elevated erythrocyte sedimentation rate (ESR) and higher angiopoietin-2/-1 ratios but lower matrix metalloproteinase (MMP)-3 levels identify concomitant GCA in PMR patients. In addition, we confirmed that elevated platelet counts are characteristic of GCA but not of GCA mimicry controls and that low MMP-3 and proteinase 3 (PR3) levels may help to discriminate GCA from infections.

**Conclusion:**

This study, performed in two independent international cohorts, consistently shows the potential of angiopoietin-2/-1 ratios and MMP-3 levels to identify GCA in patients presenting with PMR. These biomarkers may be used to select which PMR patients require further diagnostic workup. Platelet counts may be used to discriminate GCA from GCA look-alike patients.

**Supplementary Information:**

The online version contains supplementary material available at 10.1186/s13075-022-02754-5.

## Key messages

What is already known about this subject?

- There is yet no single symptom, physical sign or laboratory test that can confirm or exclude the diagnosis of GCA.

What does this study add?

- The angiopoietin-2/1 ratio and MMP-3 levels in serum of patients presenting with PMR symptoms may aid the diagnosis of concomitant GCA.
- Platelet counts may be the best biomarker to discriminate GCA patients from patients with look-alike conditions.

How might this impact on clinical practice or future developments?

- Screening for these markers could aid the decision to start further diagnostic workup including imaging.

## Introduction

Giant cell arteritis (GCA) is a seriously debilitating disease affecting people over 50 years old [1]. Cranial symptoms of GCA including headache, jaw claudication and vision loss [1, 2] are disease-specific symptoms. But the majority of GCA patients suffer from systemic symptoms like fever, fatigue, weight loss and night sweats. Moreover, GCA commonly overlaps with polymyalgia rheumatica (PMR), a systemic inflammatory disease caused by inflammation of mainly the shoulders and hips. The reported incidence of GCA among PMR patients varies between 16 and 21% [1]. Difficulties in recognising GCA do not only occur among patients presenting with PMR symptoms; also patients presenting with infectious symptoms can in fact have GCA [3, 4]. Early recognition of GCA is crucial in order to detect and prevent irreversible damage such as vision loss and aortic aneurysms in time [5, 6]. However, this is challenging, as presently no single symptom, physical sign or laboratory test can confirm or exclude the diagnosis of GCA [7].

Symptoms of GCA are caused by infiltration of immune cells into the vascular wall of medium-sized and large arteries, often also leading to systemic inflammation [8]. We previously reported on an expansion of circulating myeloid cell counts, monocytes and neutrophils, in treatment-naïve GCA and PMR patients [9]. Other important processes in GCA pathology are tissue destruction caused by matrix metalloproteases (MMPs) and the formation of new endothelial vessels in the vessel wall (neoangiogenesis) [8, 10, 11].

PMR patients are commonly treated by their general practitioner, who typically has limited, if any, means to exclude overlapping GCA by imaging. Given that GCA patients are at risk of developing serious vascular complications [5] and that they require a substantially higher glucocorticoid (GC) dose, we previously sought out new biomarkers that can identify subclinical GCA among patients presenting with PMR clinic [12]. This single-centre study pointed at the possible diagnostic utility of the angiopoietin-2/angiopoietin-1 ratio. So far, many biomarkers (e.g. C-reactive protein (CRP)) are found to be elevated in GCA and PMR when compared to healthy controls (HCs) but not when compared to infectious controls or look-alikes [13].

Thus, disease-specific diagnostic biomarkers are lacking for GCA, a disease in which early recognition is key. Despite their obvious value in the diagnostic workup for GCA, imaging techniques are costly and often unavailable in daily clinical practice, and during the pandemic, the access to imaging modalities became even more difficult [3, 4, 14]. With this study, we aim to answer two research questions. The first is whether we can validate markers that can detect GCA in patients presenting with PMR. The second is whether these markers can discriminate between GCA and look-alikes mimicking GCA. Based on our previous research, we selected promising biomarkers for the detection of GCA. We investigated the selected biomarkers, together with disease symptoms, in two independent international cohorts.

## Methods

### Cohort inclusion and exclusion

This study comprises clinical and laboratory data of GCA patients, PMR patients, HCs, and disease control groups in two independent prospective cohorts (Table 1). No participant was using immunosuppressive drugs, such as GCs, at the time of inclusion.

**Table 1:** Patient characteristics of HCs, GCA patients, PMR patients and disease control groups in both cohorts

		Aarhus cohort				Groningen GPS cohort			
		HC	GCA	Isolated PMR	GCA look-alike	HC	GCA	Isolated PMR	Infection control
*n*		19	52	25	18	45	48	39	16
Age	Years	66	67	68	70	71	72	73	73
Sex	% female	68	62	52	39	62	69	59	35
**Classification criteria**									
ACR for GCA	% pos	0	85	8	44	0	71	15	NA
Chuang for PMR	% pos	0	19	68	0	0	15	72	NA
ACR/EULAR for PMR	% pos	0	8	68	11	0	8	85	NA
**Diagnostic tools**									
TAB GCA	pos/neg/NA	NA	36/12/4	0/23/2	0/11/7	NA	23/7/18	0/7/32	NA
PET GCA	pos/neg/NA	NA	48/4/0	0/25/0	0/13/5	NA	32/5/11	0/29/10	NA
US GCA	pos/neg/NA	NA	47/5/0	2/23/0	1/23/1	NA	25/17/6	1/9/39	NA
**Symptoms**									
New headache	%	NA	60	12	50	NA	75	23	NA
Jaw/tongue claudication	%	NA	21	0	6	NA	42	13	NA
Abnormal temporal artery	%	NA	27	0	22	NA	50	8	NA
Visual symptoms	%	NA	4	0	22	NA	29	0	NA
Scalp tenderness	%	NA	33	8	28	NA	46	10	NA
Limb claudication	%	NA	19	8	0	NA	19	15	NA
Fever	%	NA	58	32	33	NA	33	15	NA
Weight loss	%	NA	85	44	33	NA	63	49	NA
Night sweats	%	NA	68^a^	36	33	NA	48	38	NA
Malaise	%	NA	92	92	39	NA	75	85	NA
Overlapping PMR	%	NA	25	NA	NA	NA	23	NA	NA
Symptom duration, median	days	NA	88	56	NA	NA	47	114	NA

^a^In the Aarhus cohort, the presence or lack of night sweats was not recorded in 8 GCA patients

The AGP cohort consists solely of a consecutive series of patients with a suspicion of GCA. A more detailed description of this cohort has previously been published [9, 15]. Patients underwent an extensive diagnostic investigation: full history taking, clinical examination, extensive laboratory analysis, temporal artery biopsy (TAB), 18F-fluorodeoxyglucose positron emission tomography-computed tomography (FDG PET/CT), and vascular ultrasound (US) imaging. Based on the final clinical diagnosis, GCA suspected patients were grouped into three categories: GCA-verified patients, PMR patients, and inflammatory controls. The GCA diagnosis was in all cases verified by either a positive TAB and/or FDG-PET-CT. Importantly, all participating patients were required to undergo FDG PET/CT imaging before treatment initiation, and patients were excluded if this was not safe.

The GPS cohort in Groningen comprises a consecutive series of patients suspected of GCA or PMR, patients with confirmed infections, and HCs. GCA diagnosis was based on the clinician’s expert opinion and either a positive TAB or FDG PET/CT. PMR diagnosis was based on the clinician’s expert opinion and often aided by FDG PET/CT imaging. PMR patients that were suspected of GCA due to suspicious cranial symptoms underwent a more intensive diagnostic workup, and all underwent a FDG PET/CT with additionally either a TAB or US imaging.

In both cohorts, we selected an important subgroup of patients with clinical symptoms of PMR (pain/stiffness of the shoulders or hips; ‘PMR clinic’). These patients subsequently received a diagnosis of overlapping GCA/PMR or isolated PMR.

GCA and PMR patients of both cohorts were compared to disease control groups. In Aarhus, samples from GCA look-alike patients were analysed. These patients were either enrolled in the AGP or the GPS cohort, but finally did not receive a diagnosis of GCA or PMR. They were diagnosed with infections, atherosclerosis, chronic kidney insufficiency, central vein occlusion, or polyarthritis. Samples from the GPS cohort were compared with a population of age-matched infection controls. These were hospitalised patients that were diagnosed with pneumonia or a severe urinary tract infection and were consecutively included in our cohort. Importantly, these patients did not suffer from other underlying diseases such as cancer or autoimmune diseases.

To put biomarker levels in perspective to control values, both cohorts of GCA and PMR patients were also compared with age- and sex-matched HCs. These HCs were recruited at the GPS cohort and were screened for past and current morbidities by a physician or specialised nurse.

### Biomarker measurements

Blood samples were drawn from patients before initiation of treatment. Serum and plasma samples were stored at −20°C (GPS) or −80°C. Whereas the Aarhus samples only went through one freeze-thaw cycle, most GPS samples underwent two or three freeze-thaw cycles. Values of CRP, ESR, leukocyte counts, and platelet counts were assessed in the context of standard medical care.

In both cohorts, serum or plasma levels of selected biomarkers, e.g. vascular endothelial growth factor (VEGF), angiopoietin-1, angiopoietin-2, soluble Tie2 (sTie2), YKL-40, MMP-3, MMP-9, soluble CD206 (sCD206), calprotectin, proteinase 3 (PR3), elastase, alpha-1 antitrypsin (A1AT), were measured by ELISA or Human premix Magnetic Luminex (Austin, TX, USA) screening assay kits. Supplementary Table S1 shows technical details of each assay. Levels of VEGF, angiopoietin-1, angiopoietin-2, sTie2 and YKL-40 in the GPS cohort have previously been measured with the same assays and published [12, 16]. They are shown here as a comparison to the Aarhus cohort data.

### Statistics

We used non-parametric testing (2-tailed) to compare the study groups. In case of a significant (*p*<0.05) Kruskal-Wallis test, specific groups were tested by the Mann-Whitney *U* test. Additionally, receiver operator characteristic (ROC) curves were used to evaluate the discriminatory performance of the markers. In addition to the area under the curve (AUC), the optimal cut-off points were calculated according to the Youden index. Analyses were performed with GraphPad Prism 8.4.2 software.

## Results

### GPS cohort and AGP cohort baseline characteristics

Baseline demographical and clinical characteristics of both the GPS cohort and the AGP cohort are displayed in Table 1. Forty-eight GCA patients and 39 PMR patients of the GPS cohort were recruited, all treatment-naïve. Two control groups were added: 45 HCs and 16 infection controls. From the AGP cohort, 52 GCA patients and 25 PMR patients, also treatment-naïve, were included. We added 19 additional HCs (transferred from the GPS cohort) and 18 GCA look-alike patients (of which 10 were transferred from the GPS cohort) as a comparison for the Aarhus GCA and PMR patients.

Cranial GCA findings, such as jaw claudication (Fisher exact test *p*=0.03), TAB abnormality (*p*=0.02), and visual disturbance (*p*=0.0007), were more common in the GPS cohort than in the AGP cohort. In contrast, systemic GCA symptoms, such as fever (*p*=0.02), weight loss (*p*=0.01), and malaise (*p*=0.03), were more common in the AGP cohort. No differences were found in clinical characterisation of PMR patients between both cohorts. Also, no differences were found in age or sex (*p*>0.05) between the cohorts.

Laboratory values for both cohorts, analysed in this study and in the context of standard care, are displayed in Table 2. In general, GCA patients in the Aarhus cohort showed evidence of a stronger inflammatory response than in the GPS cohort (i.e. a trend for higher CRP and platelet count). This was also true for the PMR patients in the Aarhus cohort as evidenced by a significantly higher platelet count and a trend to a higher leukocyte count (see Supplementary Table S2 for cohort differences).

**Table 2:** Biomarker concentrations for the study groups in both cohorts

		Aarhus cohort				Groningen GPS cohort			
		HC	GCA	Isolated PMR	GCA look-alike	HC	GCA	Isolated PMR	Infection control
CRP, median (IQR)	mg/L	1.1 (0.5–2.9)	**74 (44**–**102)**	**35 (23**–**73)**	54 (13–107)	0.7^a^ (0.5–2.4)	**52 (19**–**97)**	**35 (12**–**67)**	70 (35–107)
ESR, median (IQR)	mm/h	8 (4–14)	**73 (63**–**91)**	**53 (33**–**70)**	75 (32–95)	9 (5–14)	**88 (50**–**104)**	**55 (36**–**72)**	60^b^ (18–109)
Leukocytes, median (IQR)	10^9^/L	5.3 (4.6–6)	**9.1 (7.6**–**10.3)**	**9.5 (8.7**–**10.8)**	8.4 (7.5–9.9)	6.1 (5.2–7.3)	**9.1 (7.2**–**11.3)**	**8.7 (7.2**–**10.2)**	NA
Platelets, median (IQR)	10^9^/L	235 (214–263)	**423 (353**–**493)**	**401 (329**–**472)**	337 (275–433)	240 (214–276)	**364 (301**–**472)**	**321 (278**–**383)**	275 (199–315)
VEGF, median (IQR)	pg/mL	90 (10–183)	145 (53–334)	160 (58–318)	237 (149–326)	*75 (52*–*143)*	***125 (71***–***269)***	***190 (124***–***239)***	*162 (66*–*280)*
Angpt-1, median (IQR)	ng/mL	67 (55–81)	**55 (33**–**68)**	65 (50–93)	67 (43–96)	*48 (41*–*60)*	*54 (47*–*67)*	*48 (39*–*66)*	*64 (47*–*81)*
Angpt-2, median (IQR)	pg/mL	1060 (960–1765)	**2892 (2059**–**4101)**	**2557 (2253**–**4094)**	2145 (1758–3748)	*952 (616*–*1570)*	***3877 (2158***–***5610)***	***1848 (1552***–***3028)***	*4417 (1878*–*7832)*
sTie2, median (IQR)	ng/mL	14 (12–16)	16 (13–21)	**18 (13**–**24)**	17 (13–20)	*10 (7*–*12)*	***14 (9***–***18)***	***12 (9***–***14)***	*13 (12*–*18)*
YKL-40, median (IQR)	ng/mL	53 (34–75)	**74 (44**–**125)**	**76 (55**–**131)**	63 (35–140)	*52 (38*–*80)*	***101 (59***–***148)***	***128 (75***–***165)***	*106 (36*–*162)*
MMP-3, median (IQR)	ng/mL	14 (11–21)	15 (10–22)	**38 (28**–**54)**	22 (13–29)	11 (8–16)	10^c^ (8–16)	**24**^**c**^**(16–49)**	27^c^ (18–39)
MMP-9, median (IQR)	ng/mL	234 (172–415)	238 (114–341)	320 (197–339)	317 (222–802)	239 (173–303)	**391**^**c**^**(239–557)**	245^c^ (198–377)	130^c^ (73–253)
sCD206, median (IQR)	ng/mL	105 (92–145)	**210 (171**–**267)**	**173 (137**–**237)**	255 (191–383)	125 (107–143)	**180 (139**–**236)**	**145 (127**–**212)**	196 (124–233)
Calprotectin, median (IQR)	ng/mL	1395 (942–1802)	**3310 (2537**–**4212)**	**4200 (2675**–**5203)**	3505 (2801–6980)	2712 (2039–4168)	**7028 (3691**–**10052)**	**5862 (4858**–**7692)**	6774 (4606–8456)
PR3, median (IQR)	ng/mL	22 (15–28)	**34 (26**–**45)**	**37 (25**–**43)**	41 (24–53)	38 (31–54)	**54 (43**–**74)**	**57 (42**–**71)**	135 (70–214)
Elastase, median (IQR)	ng/mL	69 (59–95)	103 (91–141)	**133 (84**–**150)**	115 (89–197)	110 (98–142)	122 (80–172)	**137 (116**–**182)**	253 (157–366)
A1AT, median (IQR)	mg/mL	1.7 (1.6–2.0)	**3.5 (2.8**–**4.0)**	**3.0 (2.6**–**3.8)**	3.0 (2.5–3.6)	1.6 (1.4–1.8)	**3.5 (2.3**–**4.4)**	**2.7 (2.3**–**3.7)**	3.8 (2.5–4.9)

Significantly (*p*<0.05) higher biomarker levels in GCA and PMR patients when compared to HCs are indicated in bold. GPS data on VEGF, angiopoietin-1, angiopoietin-2, sTie2 and YKL-40 have previously been published and are shown here as a reference (indicated in italics) [12, 16]

*IQR* Interquartile range

^a^The HC CRP median in the GPS cohort shown here is the median of 9 HCs. CRP concentrations in the remaining 36 HCs were all lower than 5 mg/L, but could not be specified further

^b^Infection control ESR levels are missing for 7 participants

^c^In the GPS cohort, MMP-3 and MMP-9 levels are missing in 5 GCA patients, 6 PMR patients and 3 infection controls

### Multiple biomarkers of inflammation elevated in patient and disease control groups

Levels of CRP, ESR, angiopoietin-2, YKL-40, calprotectin, sCD206, PR3, A1AT, and platelet and leukocyte counts were upregulated in all patient groups of both cohorts when compared to HCs (Table 2). In both cohorts, MMP-3 levels were upregulated in PMR patients and disease control groups, but not in GCA patients.

### Factors that identify concomitant GCA in patients presenting with PMR

We evaluated clinical or biological factors that could flag overlapping GCA in PMR patients in both cohorts. To this end, we studied patients presenting with symptoms of PMR (PMR clinic), which amounted to 38 in the AGP cohort and 50 in the GPS cohort. Within these groups, 13 and 11 patients respectively had FDG PET-CT-proven GCA.

**Fig. 1:**
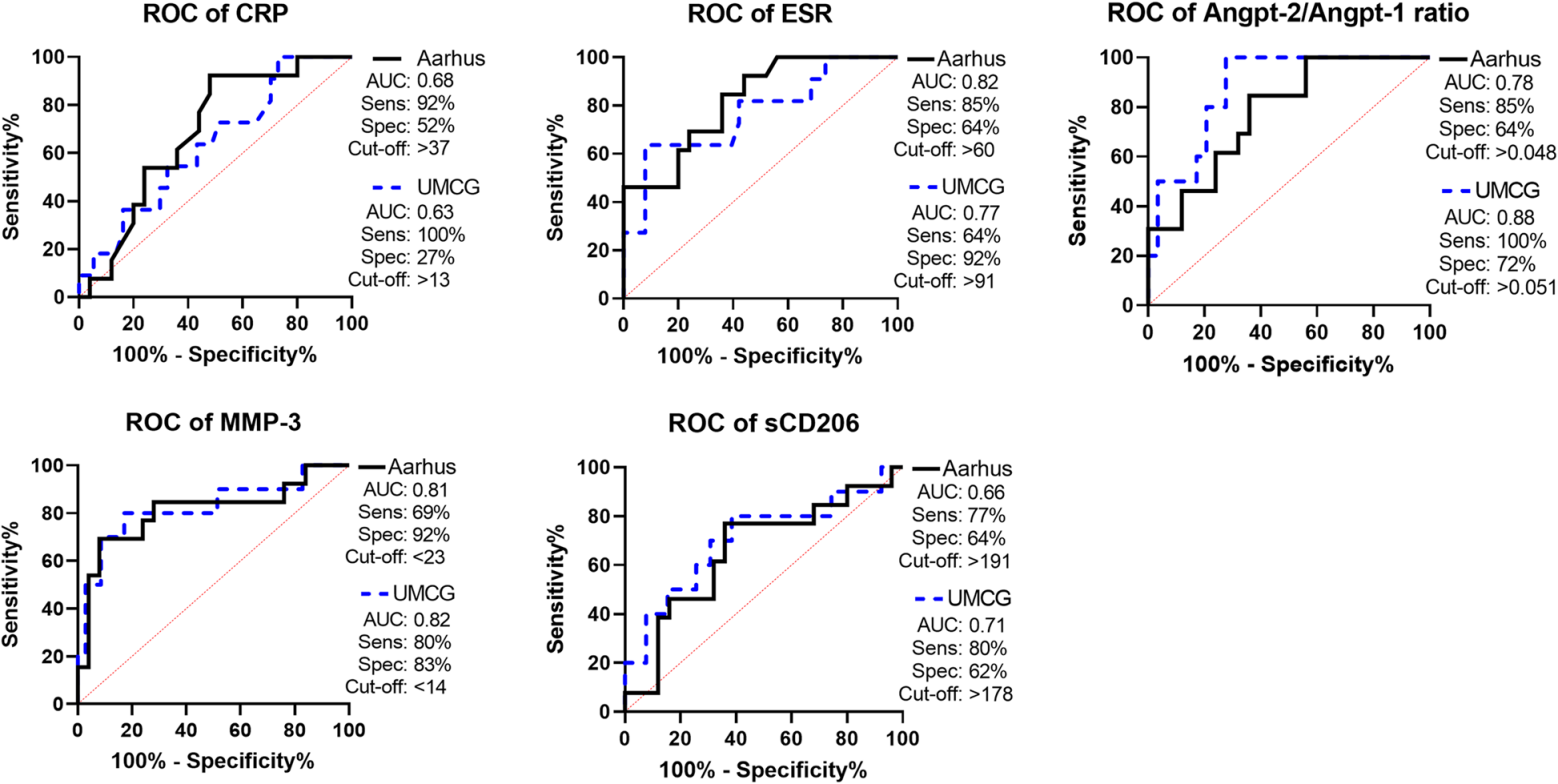
ROC curves for biomarker levels in overlapping GCA/PMR patients as compared to isolated PMR patients. Shown are ROC curves in solid black for the Aarhus cohort and dotted blue for the GPS cohort and the corresponding values of the area under the curve (AUC). Optimal sensitivity (Sens), specificity (Spec) and cut-off values were calculated according to the Youden index. In the Aarhus cohort, overlapping GCA/PMR *N*=13 and isolated PMR *N*=25. In the GPS cohort, *N*=11 for overlapping GCA/PMR and *N*=39 for isolated PMR, except for angpt-2/angpt-1 ratio (*N*=10 and 29, respectively) and MMP-3 (*N*=10 and 35, respectively). ROC receiver operating characteristic

Five biomarkers consistently discriminated between GCA/PMR overlap patients and isolated PMR patients in both cohorts (Fig. 1). In line with our previous report [12], a high ESR and a high angiopoietin-2/angiopoietin-1 ratio outperformed CRP as an identifier for overlapping GCA/PMR. Moreover, low levels of MMP-3 were also found to be an excellent identifier of concomitant GCA in PMR patients. AUCs and additional ROC data for all biomarkers are displayed in Supplementary Table S3.

In both cohorts, the presence of weight loss at diagnosis signalled overlapping GCA in patients presenting with PMR symptoms (Table 3). Only a minority of isolated PMR patients presented with weight loss at diagnosis, whereas in overlapping GCA/PMR weight loss was recorded in all but one patient (in the Aarhus cohort, *p*=0.005) or even all patients (in the GPS cohort, *p*=0.02).

**Table 3:** Cranial and systemic symptoms differ in overlapping GCA/PMR patients when compared to isolated PMR patients

		Aarhus cohort			Groningen GPS cohort		
		GCA/PMR overlap	Isolated PMR	***p***-value	GCA/PMR overlap	Isolated PMR	***p***-value
*N*		13	25		11	39	
New headache	%	54	12	0.02	45	23	ns
Jaw/tongue claudication	%	23	0	0.03	15	13	ns
Abnormal temporal artery	%	31	0	0.0097	8	8	ns
Visual symptoms	%	0	0	ns	23	0	0.008
Scalp tenderness	%	15	8	ns	15	10	ns
Limb claudication	%	31	8	ns	23	15	ns
Fever	%	54	32	ns	31	15	ns
Weight loss	%	92	44	0.005	85	49	0.0016
Night sweats	%	75	36	0.04	46	38	ns
Malaise	%	100	92	ns	62	85	ns

In both cohorts, the incidence of symptoms was compared between the two patient populations using Fisher’s exact test. The only symptom that is significantly more common in overlapping GCA/PMR patients of both cohorts is weight loss. Of note, weight loss is scored as >2 kg in the GPS cohort and >3 kg in the Aarhus cohort

### Factors that discriminate GCA patients from disease controls

We compared, in the AGP cohort, patients with a definitive diagnosis of GCA with patients that were suspected of GCA, but eventually received a different diagnosis. In the GPS cohort, we compared patients with a definitive diagnosis of GCA with infectious controls.

**Fig. 2:**
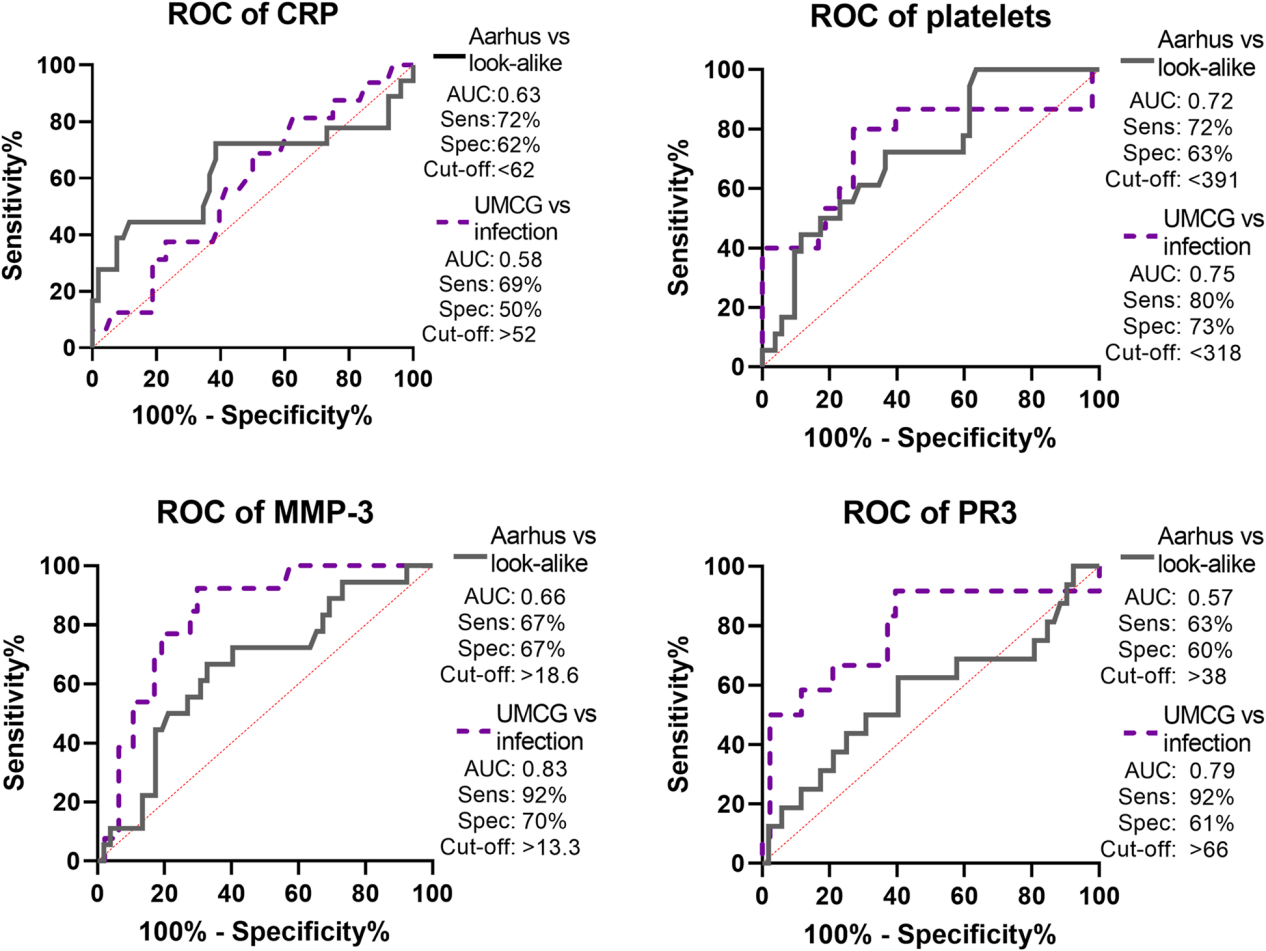
ROC curves for GCA patients as compared to non-GCA disease control groups. In the Aarhus cohort, biomarker levels were compared between treatment-naïve GCA patients (solid grey, *N*=52) and patients who were suspected of GCA, but received a different diagnosis (look-alike, *N*=18). In the GPS cohort, treatment-naïve GCA patients (dotted purple, *N*=48) were placed against infection controls (*N*=16). In addition to the area under the curve (AUC), optimal sensitivity (Sens), specificity (Spec) and cut-off values were calculated according to the Youden index. ROC receiver operating characteristic

In the AGP cohort, we validated that high platelet counts are a disease-specific biomarker of GCA, discriminating GCA from look-alikes and infectious controls (Fig. 2). Low serum MMP-3 and PR3 levels could also possibly aid in the discrimination between GCA patients and infectious controls. AUCs and additional ROC data for all biomarkers are displayed in Supplementary Table S4.

Clinically, we compared the GCA look-alike population with the GCA patient populations in both cohorts. The systemic symptoms weight loss and malaise were significantly more common in GCA patients in both cohorts compared to the look-alike patients. Cranial symptoms were more common in GCA patients in the GPS cohort than the look-alike group, with significantly more jaw claudication and a trend to more headache and an abnormal temporal artery.

## Discussion

**Fig. 3:**
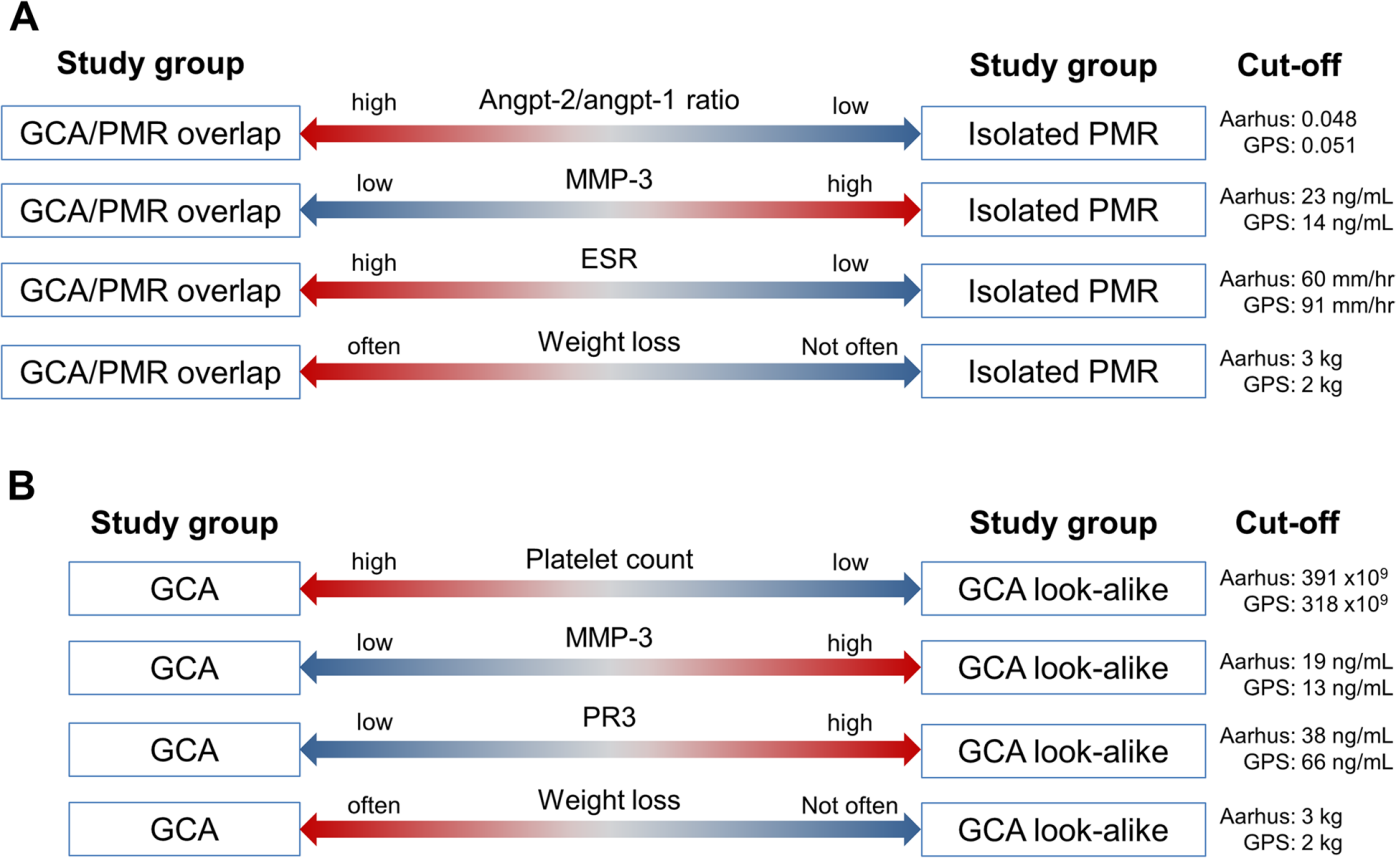
Summary of the most important and consistent findings in both cohorts. **A** The four factors that perform best in discriminating GCA/PMR patients overlap from isolated PMR patients in both cohorts. **B** The four factors that perform best in discriminating GCA patients from GCA look-alike patients in both cohorts. Cut-off values for the biomarkers are calculated by the Youden index

Distinguishing GCA from isolated PMR and other look-alike conditions remains difficult in the daily clinical practice. Only a minority of patients suspected of GCA will typically receive a GCA diagnosis [17]. Given that imaging and biopsies can be invasive, time-consuming, costly, and difficult to organise especially during the pandemic, new tools are required to increase the probability of a GCA diagnosis. Therefore, we investigated the diagnostic utility of disease-specific biomarkers and recorded clinical symptoms. This study is among the first to assess the diagnostic value of selected biomarkers in two independent international prospective cohorts of GCA patients. We identified three biomarkers that perform best in discriminating GCA/PMR from isolated PMR, as well as GCA from (infectious) look-alikes (Fig. 3). This study validated previous data [7, 9, 12, 16, 18] and achieved two important goals: detecting GCA in patients presenting with PMR clinic and discriminating GCA patients from patients with mimicking conditions.

The most promising markers for detecting vascular inflammation in patients presenting with PMR are a high angpt-2/angpt-1 ratio and low MMP-3 levels. All patients presenting with PMR symptoms can be screened for angpt-2/angpt-1 ratio and MMP-3 using commercially available assays. A decision then could be made whether further diagnostic workup needs to be performed, such as TAB, US, or FDG-PET-CT to rule in or rule out GCA. In case performing these tests in all PMR patients would not be feasible, it could be considered using these tests in patients presenting with symptoms suggestive of GCA, including weight loss.

High platelet counts, possibly in conjunction with low levels of MMP-3 and PR3, discriminate GCA patients from look-alikes or infectious controls in this study. Platelets are released into the circulation in sustained inflammatory conditions, mainly via IL-6 signalling [19]. However, CRP levels are also dependent on IL-6 [16], and we show a similar CRP for GCA and GCA look-alike patients. In acute inflammatory conditions, i.e. infections, platelet counts can drop substantially before restoring within a few days [20]. The higher platelet counts may therefore reflect a longer-lasting inflammatory condition in GCA patients. Remarkably, we previously reported that platelet counts remained elevated in GCA patients even in sustained treatment-free remission [9]. In addition to biomarkers, weight loss and malaise may add to previously described symptoms of jaw and limb claudication as clinical warning markers for GCA [7].

The markers used in this study were selected for their association with pathogenic processes in GCA. As neutrophil and monocyte counts are higher in GCA and PMR [9], our finding of elevated levels of their soluble products in the blood, such as YKL-40, sCD206, calprotectin and PR3, are not unexpected [21–24]. The different angpt-2/angpt-1 ratio in overlapping GCA/PMR compared to isolated PMR likely points at essential variances in neoangiogenic expansions of the vasa vasorum in the vessel wall compared to those in the synovia. The elevated levels of MMP-3 in PMR patients compared to GCA patients have been reported before [18]. Possibly, MMP-9 production is more pronounced in GCA lesions than in PMR lesions, a process that consumes MMP-3 [25, 26]. Alternatively, the serum MMP-3 levels may reflect a more extensive synovial inflammation in isolated PMR patients than in overlapping GCA/PMR patients. Indeed, synovia affected by rheumatoid arthritis are known to release high levels of MMP-3 into the circulation. PMR synovial biopsy studies should reveal whether these tissues are also a rich source of MMP-3, like their rheumatoid arthritis counterparts [27].

Our confidence in these biomarker data is high, as they are retrieved from measurements in two independent cohorts that vary from each other in a few notable characteristics. Likely, the requirement of a treatment-naïve FDG-PET-CT scan for the inclusion in the Aarhus cohort has led to the exclusion of a number of cranial GCA patients, to prevent visual complications. This bias towards more systemic GCA may explain the stronger acute-phase response observed in the Aarhus cohort [16]. This bias may also have led to the observed differences in absolute biomarker levels between both cohorts. In addition, PMR patients in the Aarhus cohort enrolled with a suspicion of GCA, whereas PMR patients in the GPS cohort did not per se. However, dissimilar laboratory conditions may also have influenced these differences.

Despite the widespread use of the CRP and ESR, very few studies have investigated additional biomarkers aiding in the diagnosis of GCA. We propose the use of angpt-2/angpt-1 ratio and MMP-3 in the workup of PMR patients and in patients with low probability GCA. A limitation of this study is the relatively small number of GCA/PMR overlap patients in both cohorts.

In conclusion, this study provides robust evidence for more disease-specific biomarkers that may substantially improve diagnostic procedures for GCA patients.

## Supplementary Information

**Additional file 1: Supplementary Table S1.** Technical details of the assays used to detect the biomarkers in the Aarhus cohort and the GPS cohort. **Supplementary Table S2.** Cohort differences in biomarkers concentrations of GCA and PMR patients. **Supplementary Table S3.** ROC analyses for biomarker levels in overlapping GCA/PMR patients as compared to isolated PMR patients. **Supplementary Table S4.** ROC analyses for biomarker levels for GCA patients as compared to non-GCA disease control groups.

## Data Availability

Individual participant data that underlie the results reported in this article are available upon reasonable request, immediately following publication. Proposals should be directed to y.van.sleen@umcg.nl.
