# Supplementary material for: Angiopoietin-2/-1 ratios and MMP-3 levels as an early warning sign for the presence of giant cell arteritis in patients with polymyalgia rheumatica
Source: Arthritis Res Ther. 2022 Mar 7;24:65. doi: 10.1186/s13075-022-02754-5 (PMC8900446; doi:10.1186/s13075-022-02754-5)
Supplement: Supplementary file 1 — Additional file 1: Supplementary Table S1. Technical details of the assays used to detect the biomarkers in the Aarhus cohort and the GPS cohort. Supplementary Table S2. Cohort differences in biomarkers concentrations of GCA and PMR patients. Supplementary Table S3. ROC analyses for biomarker levels in overlapping GCA/PMR patients as compared to isolated PMR patients. Supplementary Table S4. ROC analyses for biomarker levels for GCA patients as compared to non-GCA disease control groups. [file 13075_2022_2754_MOESM1_ESM.docx]

|  | **Cohort** | **Method** | **Company** | **Lower limit (pg/mL)** | **Upper limit**  **pg/mL** | **Sensitivity**  **pg/mL** | **Sample type** | **Dilution** |
| --- | --- | --- | --- | --- | --- | --- | --- | --- |
| **VEGF** | Aarhus | ELISA | R&DSystems,  Abingdon, UK | 15.6 | 1000 | 9.0 | Serum | 1:2 |
| **Angpt-1** | Aarhus | Luminex | R&Dsystems | 114 | 27,61 | 9.43 | Serum | 1:50 |
| **Angpt-2** | Aarhus | Luminex | R&Dsystems | 91 | 22,000 | 17.1 | Serum | 1:2 |
| **sTie2** | Aarhus | Luminex | R&Dsystems | 670 | 162,700 | 211 | Serum | 1:2 |
| **YKL-40** | Aarhus | Luminex | R&Dsystems | 352 | 85,610 | 3.30 | Serum | 1:2 |
| **MMP-3** | Both | Luminex | R&Dsystems | 83 | 20,159 | 5.3 | Serum | 1:2 |
| **MMP-9** | Both | Luminex | R&Dsystems | 134 | 32,596 | 13.6 | Serum | 1:50 |
| **sCD206** | Both | ELISA | Hycult Biotech, Uden, The Netherlands | 3,100 | 200,000 | <3,100 | Serum | 1:5 |
| **Calprotectin** | Both | ELISA | Hycult | 1,600 | 100,000 | <1,600 | Serum | 1:200 |
| **PR3** | Both | ELISA | Hycult | 630 | 40,000 | <630 | Plasma | 1:10 |
| **Elastase** | Both | ELISA | Hycult | 400 | 25,000 | <400 | Plasma | 1:30 |
| **A1AT** | Both | ELISA | Hycult | 1,600 | 100,000 | <1,600 | Serum | 1:160,000 |

***Supplementary Table S1:*** *Technical details of the assays used to detect the biomarkers in the Aarhus cohort and the GPS cohort. In Aarhus, the Luminex tests were read on a Luminex Magpix instrument (Luminex, Austin, TX, USA) and ELISA tests on a FLUOstar Omega reader (BMG Labtech, Ortenberg, Germany). For the GPS cohort, the Luminex test were read on a Luminex Magpix instrument and ELISA tests on a VERSAmax reader (Molecular Devices, San Jose, CA, USA).*

| **Aarhus cohort compared to GPS cohort** | | |  | | |  |  | | |  |
| --- | --- | --- | --- | --- | --- | --- | --- | --- | --- | --- |
|  | **GCA** | **PMR** |  |  | p= | | |  |  |  |
| CRP | # |  |  | # | 0.05-0.10 | | |  |  |  |
| ESR |  |  |  | * | 0.01-0.05 | | |  |  |  |
| Leukocytes |  | # |  | ** | 0.001-0.01 | | |  |  |  |
| Platelets | # | ** |  | *** | <0.001 | | |  |  |  |
| Hb | ** |  |  |  |  | | |  |  |  |
| VEGF |  |  |  | Red | Aarhus higher than GPS | | |  |  |  |
| Angpt-1 |  | * |  | Blue | Aarhus lower than GPS | | |  |  |  |
| Angpt-2 | # | * |  |  |  | | |  |  |  |
| sTie-2 |  | *** |  |  |  | | |  |  |  |
| YKL-40 |  | * |  |  |  | | |  |  |  |
| MMP-3 | ** | # |  |  |  | | |  |  |  |
| MMP-9 | *** |  |  |  |  | | |  |  |  |
| sCD206 | * |  |  |  |  | | |  |  |  |
| Calprotectin | *** | *** |  |  |  | | |  |  |  |
| PR3 | *** | *** |  |  |  | | |  |  |  |
| Elastase |  | # |  |  |  | | |  |  |  |
| A1At |  |  |  |  |  | | |  |  |  |

***Supplementary Table S2:*** *Cohort differences in biomarkers concentrations of GCA and PMR patients. Even though the biomarker assays are the same, the Mann Whitney U test reveals significant differences when comparing patients from the GPS cohort with patients from the Aarhus cohort*

|  | **Aarhus GCA/PMR overlap vs isolated PMR** | | | | **GPS GCA/PMR overlap vs isolated PMR** | | | |
| --- | --- | --- | --- | --- | --- | --- | --- | --- |
|  | **AUC** | **Sens (%)** | **Spec (%)** | **Cut-off** | **AUC** | **Sens (%)** | **Spec (%)** | **Cut-off** |
| CRP | 0,68 | 92 | 52 | >37 mg/L | 0,63 | 100 | 27 | >13 mg/L |
| ESR | 0,82 | 85 | 64 | >60 mm/hr | 0,77 | 64 | 92 | >91 mm/hr |
| platelets | 0,66 | 62 | 76 | >467*10^9^ cells | 0,67 | 46 | 87 | >435*10^9^ cells |
| VEGF | 0,51 | 46 | 68 | >254 pg/mL | 0,66 | 70 | 76 | <122 pg/mL |
| Angiopoietin-1 | 0,71 | 38 | 100 | <35 ng/mL | 0,5 | 70 | 52 | >49 ng/mL |
| Angiopoietin-2 | 0,7 | 85 | 68 | >3051 pg/mL | 0,9 | 100 | 76 | >3124 pg/mL |
| Angpt-2/Angpt1 ratio | 0,78 | 85 | 64 | >0,048 | 0,9 | 100 | 76 | >0,051 |
| sTie2 | 0,6 | 62 | 64 | <17 ng/mL | 0,74 | 70 | 83 | >16 ng/mL |
| YKL-40 | 0,55 | 31 | 84 | <50 ng/mL | 0,51 | 40 | 72 | <93 ng/mL |
| MMP-3 | 0,81 | 69 | 92 | <23 ng/mL | 0,82 | 80 | 83 | <14 ng/mL |
| MMP-9 | 0,68 | 46 | 96 | <137 ng/mL | 0,62 | 50 | 80 | >385 ng/mL |
| Calprotectin | 0,7 | 92 | 52 | <4127 ng/mL | 0,55 | 30 | 95 | >9555 ng/mL |
| sCD206 | 0,66 | 77 | 64 | >191 ng/mL | 0,71 | 80 | 62 | >178 ng/mL |
| Elastase | 0,58 | 85 | 56 | <125 ng/mL | 0,56 | 50 | 92 | <72 ng/mL |
| A1AT | 0,58 | 77 | 52 | >3,0 mg/mL | 0,5 | 50 | 69 | <2,4 mg/mL |
| PR3 | 0,5 | 85 | 40 | >28 ng/mL | 0,58 | 50 | 78 | >72 ng/mL |

**Supplementary Table S3:** *ROC analyses for biomarker levels in overlapping GCA/PMR patients as compared to isolated PMR patients. AUC: area under the curve, Sens: sensitivity, Spec: specificity.*

|  | |  | | | | |  | | | |  |
| --- | --- | --- | --- | --- | --- | --- | --- | --- | --- | --- | --- |
|  | **Aarhus GCA vs look-alike** | | | | | **GPS GCA vs infection controls** | | | | | |
|  | **AUC** | | **Sens (%)** | **Spec (%)** | **Cut-off** | **AUC** | | **Sens (%)** | **Spec (%)** | **Cut-off** | |
| CRP | 0.63 | | 72 | 62 | <62 mg/L | 0.58 | | 69 | 50 | >52 mh/L | |
| ESR | 0.55 | | 39 | 96 | <49 mm/hr | 0.58 | | 44 | 92 | <33 mm/hr | |
| platelets | 0.72 | | 72 | 63 | <391*10^9^ cells | 0.75 | | 80 | 73 | <318*10^9^ cells | |
| VEGF | 0.6 | | 83 | 50 | >139 pg/mL | 0.54 | | 54 | 64 | >163 pg/mL | |
| Angiopoietin-1 | 0.64 | | 50 | 77 | >69 ng/mL | 0.68 | | 69 | 73 | >61 ng/mL | |
| Angiopoietin-2 | 0.6 | | 50 | 79 | <2012 pg/mL | 0.57 | | 31 | 86 | >7171 pg/mL | |
| Angpt-2/Angpt-1 ratio | 0.63 | | 39 | 85 | <0.029 | 0.57 | | 54 | 69 | <0.057 | |
| sTie2 | 0.51 | | 67 | 48 | >15.6 ng/mL | 0.55 | | 92 | 38 | >10.8 ng/mL | |
| YKL-40 | 0.52 | | 39 | 75 | <45 ng/mL | 0.51 | | 54 | 58 | >104 ng/mL | |
| MMP-3 | 0.66 | | 67 | 67 | >18.6 ng/mL | 0.83 | | 92 | 70 | >13.3 ng/mL | |
| MMP-9 | 0.69 | | 83 | 46 | >213 ng/mL | 0.72 | | 77 | 77 | <219 ng/mL | |
| Calprotectin | 0.58 | | 39 | 94 | >5642 ng/mL | 0.54 | | 88 | 35 | <8882 ng/mL | |
| sCD206 | 0.61 | | 67 | 60 | >231 ng/mL | 0.5 | | 50 | 66 | >203 ng/mL | |
| Elastase | 0.58 | | 31 | 94 | >191 ng/mL | 0.52 | | 88 | 28 | >82 ng/mL | |
| A1AT | 0.65 | | 89 | 42 | <3.66 mg/mL | 0.58 | | 56 | 67 | >3.77 mg/mL | |
| PR3 | 0.57 | | 63 | 60 | >38 ng/mL | 0.79 | | 92 | 61 | >66 ng/mL | |

**Supplementary Table S4:** *ROC analyses for biomarker levels for GCA patients as compared to non-GCA disease control groups. AUC: area under the curve, Sens: sensitivity, Spec: specificity.*
